# Association Between Orthostatic Intolerance Symptoms and Cognitive Complaints in Hypermobile Ehlers-Danlos Syndrome (hEDS) and Joint Hypermobility Spectrum Disorder (JHSD): A Cross-Sectional Analysis

**DOI:** 10.7759/cureus.86123

**Published:** 2025-06-16

**Authors:** Ansa Tariq, Marium Nadeem Khan, Ayesha Ghazal Jamali, Manaal Amir Basra, Aakash Mahato, Syed Mariyum Imran, Zainab Mohsin, Shivam Singla, Imran K Mirani, Uzma Asmat, Habiba Malik

**Affiliations:** 1 Internal Medicine, Lahore Medical and Dental College, Lahore, PAK; 2 Medicine, Shifa College of Medicine, Islamabad, PAK; 3 Internal Medicine, Liaquat University of Medical and Health Sciences, Jamshoro, PAK; 4 Internal Medicine, Rahbar Medical and Dental College, Lahore, PAK; 5 Internal Medicine, Mirbat Hospital, Salalah, OMN; 6 Internal Medicine, Jinnah Sindh Medical University, Karachi, PAK; 7 Internal Medicine, Shifa College of Medicine, Islamabad, PAK; 8 Internal Medicine, TidalHealth Peninsula Regional, Salisbury, USA; 9 Internal Medicine, Via Medica International Healthcare, Abu Dhabi, ARE; 10 Medicine, Continental Medical College, Lahore, PAK

**Keywords:** autonomic dysfunction, brain fog, cognitive failure, hypermobile ehlers-danlos syndrome, orthostatic intolerance

## Abstract

Background: Many people with hypermobile Ehlers-Danlos syndrome (hEDS) and joint hypermobility spectrum disorder (JHSD) often experience both orthostatic intolerance (OI) and brain fog. They are both clinically significant, even though their direct link has rarely been studied. This research examines whether there is a link between OI symptoms and complaints of poor concentration in patients with hEDS and JHSD.

Materials and methods: A study was carried out with 385 individuals who were clinically diagnosed with hEDS or JHSD in Islamabad, Pakistan. Both the Composite Autonomic Symptom Score (COMPASS-31) and the Cognitive Failure Questionnaire (CFQ) were used to collect data. To investigate the relationship between OI problems and cognitive symptoms, a statistical analysis was conducted using SPSS Statistics version 26 (IBM Corp. Released 2019. IBM SPSS Statistics for Windows, Version 26.0. Armonk, NY: IBM Corp.), including Pearson correlation, t-tests, ANOVA, and linear regression. Data were collected from November 15, 2024, to March 25, 2025.

Results: Orthostatic symptoms were present in 249 (65%) of participants, and cognitive changes were reported by 282 (73%). The correlation between OI and CFQ scores was found to be moderate and statistically significant (r = 0.449, p = 0.03). Participants who experienced brain fog often (36%) scored a bit higher on CFQ than others. Although the differences between the groups were found to be significant, the effect sizes were very small (η² = 0.004), which corresponds to practically irrelevant distinctions. In a regression model, it was confirmed that the OI symptoms were significant predictors of cognitive complaints (r = 0.449, p = 0.03).

Conclusions: The researchers found a statistically significant yet low level of association between OI and cognitive complaints in individuals with hEDS and JHSD. These results suggest that autonomic dysfunction may play a role in the cognitive symptoms experienced by this group of individuals. Future objective assessment studies should be conducted to better understand the mechanisms involved and the contributions of psychological and emotional factors.

## Introduction

Ehlers-Danlos syndromes (EDS) refer to a set of inherited connective tissue disorders characterized by joint hypermobility, skin hyperextensibility, and tissue fragility, all of which involve multiple organ systems. Although the genetic bases have been established for most subtypes of EDS, the hypermobile type (hEDS) remains without a known molecular explanation, making it highly challenging to diagnose and treat [1,2]. hEDS is the most common type of EDS, characterized by joint hypermobility, subluxations/dislocations, and chronic pain in the musculoskeletal system. Formerly known as joint hypermobility syndrome, it is now classified under hEDS, where diagnosis continues to be clinical given the absence of a known genomic marker [3].

A study on the prevalence of hypermobility spectrum disorder (HSD) and EDS showed that the rate of these disorders was unusually high, and the point prevalence of the diagnosis was 194.2 per 100,000. It also released other comorbidities that were related to the musculoskeletal system, including mental health and gastrointestinal disorders [4]. Among gastrointestinal symptoms common in patients with hEDS and HSD are bloating, reflux, and abdominal discomfort, which, in addition to being quality-of-life-altering, are not life-threatening. Moreover, psychiatric comorbidities, including anxiety and depression, are prevalent in this group, which is probably because of the autonomic dysfunction, disturbed proprioception, and heightened emotional reactivity that are shown in neuroimaging investigations [5,6]. HSD and joint HSD (JHSD) are characterized by joint hypermobility and associated musculoskeletal symptoms. However, they are not connected with systemic symptoms, which are characteristic of hEDS. hEDS is a systemic, inherited connective tissue disorder that may also comprise chronic pain, gastrointestinal dysmotility, and manifestations such as dolichocolon and Arnold Chiari type I malformation [7]. Diagnostic criteria of hEDS are generalized joint hypermobility, soft skin, and family history [3], whereas JHSD is diagnosed by musculoskeletal complaints not involving components of systemic diseases [2]. Although HSD and JHSD are primarily associated with musculoskeletal complaints, hEDS presents more generalized symptoms that affect multiple organ systems [2,3,7]. Patients diagnosed with hEDS also present with a wide variety of symptoms, ranging from joint and limb pain, chronic fatigue, anxiety, and depression, which immensely compromise the normal functioning of the patient's day-to-day activities and quality of life. Even after several treatments, a lot of people are still experiencing constant pain and inability, indicating the difficulties in dealing with this condition [8].

hEDS is also misdiagnosed and is a condition that presents with joint instability, chronic pain, fatigue, and gastrointestinal disorders. It is the most common and the least recognized subtype of EDS with extra-articular manifestations: cardiovascular, neurological, and psychiatric disorders [7,9]. Severe abnormalities of unusual types, such as dolichocolon and Arnold-Chiari type I malformation, have also been noted, pointing to the multisystemic tendency and likelihood of disability if left uncontrolled [7]. Studies have continuously demonstrated that orthostatic intolerance (IO) is strongly related to joint hypermobility or hEDS [10]. Patients suffering from these conditions present a significantly higher rate of cardiovascular and autonomic anomalies; the detection of such anomalies may positively influence both clinical practice and research results [10].

Adults with JHSD or hEDS have high incidences of cardiovascular autonomic dysfunction. Findings include abnormal reactions, postural orthostatic tachycardia, and an increase in baroreflex sensitivity, indicating these as possible disease markers for future studies [11]. OI, including orthostatic hypotension (OH) and postural tachycardia syndrome (POTS), is a condition affecting autonomic control, resulting in symptoms such as lightheadness and fainting. The conditions may have a profound impact on quality of life, and mechanisms such as adrenergic hypofunction and redistribution of blood volume promote their development [12]. OI has been marked out as one of the leading causes of fatigue in hEDS people. Compromised cardiovascular responses to orthostatic challenges, including an elevated heart rate and decreased peripheral resistance, were highly associated with chronically measured fatigue severity [13].

People with hEDS tend to suffer from chronic and disabling pain not responsive to standard treatments in the biomedical field [14]. Emerging evidence depicts the impact of psychological factors (including increased body awareness and emotional distress, plus maladaptive activity patterns) on augmenting pain and disability, revealing the necessity of biopsychosocial management [14]. Pain, fatigue, and inability to keep up with peers are reported to be the most distressing challenges in children and adolescents with hEDS [15]. These physical problems predict functional disability, anxiety, and depression very well and point to the need for a holistic approach to care that manages physical and psychological well-being [15]. Patients with JHSD or hEDS often have psychological disorders like anxiety and depression. Possible mechanisms are autonomic dysfunction, abnormal sensory processing, and changed body awareness [6,16]. There is also emerging evidence regarding the associations with ADHD, autism spectrum disorders, and integrative psychological and physical care needs are emphasized [6,16].

Although a growing clinical recognition of such presentations exists, the specifics regarding the correlation with cognitive dysfunction remain unknown in this population. Impaired cognitive functioning in people with OI is frequently reported as deficits in concentration, memory, and mental clarity that are believed to be caused by inadequate blood flow to the brain secondary to autonomic dysfunction. Understanding this association is relevant because OI and cognitive complaints have a significant negative impact on daily functioning and quality of life, and can result in misdiagnosis or underdiagnosis in victims.

Rationale

Prior research has illustrated an increased frequency of both OI and complaints of cognitive disturbances among individuals with hEDS and JHSD. However, relatively few studies have directly explored the relationship between these two domains of symptoms. Learning about such a relationship could also facilitate more comprehensive and integrated clinical evaluation approaches. Furthermore, cognitive symptoms among these patients are often overlooked or mistakenly attributed to entirely psychological causes, resulting in undue delays in proper management. By elucidating the relationship between OI and cognitive complaints, healthcare providers can improve their ability to validate patients' experiences and tailor interventions to address both autonomic and mental concerns.

Primary objective

The primary objective of this research is to investigate the relationship between OI symptoms, as measured by the Composite Autonomic Symptom Score (COMPASS-31), and cognitive complaints, as assessed by the Cognitive Failures Questionnaire (CFQ), among individuals with hEDS or JHSD.

Secondary objective

The study will also look at the general prevalence and severity of OI symptoms and cognitive complaints in this population. Another objective is to study whether psychological and physical factors like fatigue, anxiety, and depression mediate or moderate the relationship between OI and cognitive symptoms. Finally, the study will see whether age, gender, or duration of illness act as moderators or mediators in this association.

## Materials and methods

Study design and methods

This study employed a cross-sectional study design to establish the association between symptoms of OI and cognitive complaints in adults with a diagnosis of hEDS or JHSD. The sample was drawn from a community population of patients with clinically diagnosed hEDS or JHSD, encompassing a range of symptom severities and clinical experiences.

The survey was conducted in Islamabad, Pakistan, from November 15, 2024, to March 20, 2025, and recruited respondents from outpatient physiotherapy clinics, primary healthcare centers, and online support groups. The approach provided a representative sample of adults in the locality living with hEDS and JHSD. Assessment of autonomic dysfunction and cognitive complaints was carried out by two validated instruments: COMPASS-31 [17] and CFQ [18]. This research design supported the evaluation of the potential link between OI and cognitive symptoms, contributing to the understanding of multisystem involvement in hypermobility disorders.

Sample size and technique

The infinite population formula was used to calculate the sample size for this research, as the actual population of people with hEDS or JHSD was unknown. The formula employed is:

\[ \text{Sample size} = \frac{Z^2 \cdot p (1 - p)}{d^2} \]

Here, Z is the z-score of the desired confidence level, p is the estimated prevalence, and d is the desired margin of error. For 95% confidence, the value of Z is 1.96, and the margin of error (d) has been taken as 0.05. The prevalence (p) was taken as 40.7%, based on the results of a similar study conducted in Pakistan with a similar population. Using these values in the formula, an estimated sample size of 385 was obtained [19].

Participants for the study were recruited using a convenience sampling method from outpatient physiotherapy clinics, primary healthcare centers, and online forums representing Islamabad, Pakistan. The method facilitated the recruitment of participants from diverse demographic backgrounds, as well as those with various clinical presentations of hEDS and JHSD. For eligibility in the study, the following inclusion criteria were required: (1) adults with a clinically established diagnosis of hEDS or JHSD according to the 2017 diagnostic criteria [20] and (2) participants who are able and willing to provide informed consent and fill in the self-report questionnaires. Nonetheless, 14 individuals below 18 were also involved with parental consent, since OI may impact adolescents, particularly those with hEDS or POTS. Additionally, 11 participants who had not received a formal diagnosis were recruited based on self-reported symptoms and were grouped for further assessment. Exclusion criteria were participants with diagnosed psychiatric or neurological conditions other than hEDS or JHSD that might independently influence cognitive function, including schizophrenia or major neurocognitive disorder, and those with incomplete clinical data or who refused to participate. The application of this sampling technique, combined with precise inclusion and exclusion criteria, ensured that the study population accurately represented the adult hEDS or JHSD community while minimizing confounding factors and selection bias.

Data collection tools and procedures

Information for this study was gathered with a standardized, self-report online questionnaire that measured both OI symptoms and cognitive complaints in individuals with hEDS or JHSD. The patients included in this study met the criteria of either hEDS or JHSD using the 2017 International EDS Consortium Diagnostic Criteria. hEDS was diagnosed clinically by the generalized joint hypermobility, soft skin, and family history, and molecularly when available. The diagnosis of JHSD was made according to clinical manifestations of joint hypermobility and related symptoms, according to the same criteria [20]. The survey included two validated tools: COMPASS-31 and CFQ. The researchers used COMPASS-31 to assess autonomic symptoms, specifically OI. It is a 31-item survey evaluating six areas of autonomic function: OI, vasomotor, secretomotor, gastrointestinal, bladder, and pupillomotor symptoms. The OI subscale of COMPASS-31, consisting of four items measuring symptoms of OI, including dizziness, blurred vision, and fainting, was, however, the sole measure utilized in this study to assess symptoms of OI. Items are weighted and contribute to a subscale score of 0-40, with higher scores representing more severe orthostatic symptoms. The psychometric properties of COMPASS-31 are of high value, as the instrument has a high internal consistency (Cronbach α = 0.91) as well as proven construct validity in the measurement of autonomic dysfunction. It was formulated by David M. Sletten, Guillermo A. Suarez, Phillip A. Low, Jay Mandrekar, and Wolfgang Singer in 2012 [17].

Meanwhile, CFQ was used to assess cognitive complaints. It is a 25-item questionnaire that measures the frequency of daily lapses in thinking, including forgetting, attentional lapses, and action slips. Each item on the questionnaire was rated on a 5-point Likert scale, with a total score range of 0 to 100. It was created by Broadbent, Cooper, FitzGerald, and Parkes in 1982. The total CFQ score showed excellent test-retest reliability, with a coefficient of 0.71 [18]. The demographic section captured important details about the participant, including age, sex, occupation, educational level, and any relevant comorbidities. This allowed contextualization of the clinical presentations of individuals with hEDS and JHSD.

The data were collected from in-person interviews conducted with trained personnel at physiotherapy clinics, primary care clinics, and various hospitals in Islamabad, Pakistan. The participants were required to sign an informed consent form before participating in the study.

Statistical analysis

Data was entered and analyzed with SPSS Statistics version 26 (IBM Corp. Released 2019. IBM SPSS Statistics for Windows, Version 26.0. Armonk, NY: IBM Corp.). Frequencies and percentages were computed to summarize demographic variables and COMPASS-31 and CFQ scores. The analysis led to the discovery of significant links between the severity of cognitive symptoms and autonomic dysfunction in those with hEDS and JHSD. To find relationships between specific variables, Pearson correlation was applied, while difference tests on independent groups were performed with t-tests and one-way ANOVAs. Finally, linear regression was used to determine whether the severity of autonomic symptoms helped predict cognitive complaints. Chi-square analysis was used to investigate whether there are links between different demographic and clinical categories. A statistical significance of p < 0.05 was used for all analyses.

Ethical considerations

The Institutional Review Board of Lumina Research Foundation, Islamabad, Pakistan, provided ethical approval for the present study under reference number IRB-2024-0021. Before participation, all participants received full information about the study's purpose and procedures, and then they provided written informed consent. Participation was completely voluntary, and participants could withdraw at any time without any risk or penalty. Throughout the entire research process, confidentiality was strictly maintained, ensuring that personal information was kept secure and used solely for the purpose of research.

## Results

Table 1 shows that a total of 385 participants were involved in the study. Age-wise, most of them were between 18 and 24 years old (N = 344, 89%), followed by those between 25 and 34 years old (N = 27, 7%), and those under 18 years old (N = 14, 4%). Gender-wise, most of the participants were male (N = 298, 77%), while the remaining were female (N = 87, 23%). At the level of education, the highest number had primary school education (N = 153, 40%), followed by secondary education (N = 104, 27%), no education (N = 65, 17%), and college or university education (N = 46, 12%). Few individuals held a bachelor's degree (N = 8, 2%) or a graduate or professional degree (N = 9, 2%). Most respondents were students (N = 160, 42%), followed by those who were unemployed (N = 108, 28%). The employed comprised 22% (N = 85), and 8% of the respondents specified that they were retired (N = 32).

**Table 1:** Demographic characteristics of participants (N = 385) f = frequency, % = percentage hEDS: hypermobile Ehlers-Danlos syndrome, JHSD: joint hypermobility spectrum disorder, POTS: postural tachycardia syndrome, EDS: Ehlers-Danlos syndrome, OI: orthostatic intolerance

Variable	f	%
Age	-	-
Under 18	14	4
18-24 years	344	89
25-34 years	27	7
Gender	-	-
Male	298	77
Female	87	23
Educational level	-	-
No formal education	65	17
Primary	153	40
Secondary	104	27
College/university	46	12
Bachelor’s degree	8	2
Graduate or professional degree	9	2
Employment status	-	-
Student	160	42
Employed	85	22
Unemployed	108	28
Retired	32	8
Clinical diagnosis	-	-
hEDS	124	32
JHSD	170	44
POTS	64	17
Another form of EDS	16	4
Not formally diagnosed	11	3
Symptoms of OI	-	-
Yes	249	65
No	112	29
Not sure	24	6
Formal diagnosis of a cognitive or memory-related condition	-	-
Yes	282	73
No	103	27
Currently taking medication	-	-
Yes	245	64
No	140	36
Symptoms of brain fog	-	-
Yes, frequently	140	36
Yes, occasionally	128	33
Rarely	76	20
Never	37	10
Not sure	4	1

JHSD was the most commonly clinically diagnosed condition, with 170 (44%), and very closely followed by hEDS at 124 (32%). POTS was 64 (17%), other EDS types were 16 (4%), and 11 (3%) did not indicate a formal diagnosis. In the methodology, POTS was initially included as a comorbid condition related to both hEDS and JHSD; however, it appeared as a distinct diagnosis in the results because it is a highly prevalent condition.

Concerning symptoms of OI, most of the respondents answered yes (N = 249, 65%), some answered no (N = 112, 29%), and others were not sure (N = 24, 6%). Most participants had a formal diagnosis of cognitive impairment or a memory disorder (N = 282, 73%), compared to the rest who did not (N = 103, 27%).

Of the total participants, 245 (64%) reported taking medication, while 140 (36%) stated they were not taking any medication. Lastly, on symptoms of brain fog, 140 participants (36%) said they experienced it often, 128 participants (33%) said they experienced it sometimes, 76 (20%) rarely experienced it, 37 (10%) never experienced it, and 4 (1%) were not sure.

Table 2 shows the intercorrelation between CFQ and COMPASS-31. The Pearson correlation coefficient shows a moderate positive correlation between symptoms of OI and cognitive failures (r = 0.449, p = 0.03). This indicates that as symptoms of OI rise, self-reported cognitive failures also rise. The relationship is statistically significant at the 0.05 level, indicating a significant association between cognitive impairment and autonomic dysfunction among the samples.

**Table 2:** Intercorrelations between the study variables * p < 0.05, ** p < 0.001 considered significant; correlation = Pearson correlation COMPASS-31: Composite Autonomic Symptom Score, CFQ: Cognitive Failure Questionnaire

Variable	COMPASS-31	CFQ	p
COMPASS-31	-	0.449	0.03
CFQ	0.449	-	0.03

Table 3 presents the descriptive statistics for CFQ and COMPASS-31, calculated based on 385 respondents. In the case of COMPASS-31, the average score is 8.12 and the standard deviation is 1.891. This indicates that, on average, OI was reported as mild by the participants, with variation in scores among participants. Conversely, the mean score of CFQ is 80.25 with a standard deviation of 6.448, which implies that the participants, on average, reported moderate levels of cognitive failures with some variability in their responses. These statistics will give a clearer description of the overall situation with OI and cognitive failures in the sample.

**Table 3:** Descriptive statistics for CFQ and COMPASS-31 (N = 385) SD: standard deviation, CFQ: Cognitive Failure Questionnaire, COMPASS-31: Composite Autonomic Symptom Score

Variable	Mean	SD
COMPASS-31	8.12	1.891
CFQ	80.25	6.448

Table 4 demonstrates the comparison of COMPASS-31 and CFQ scores in patients with and without the formal diagnosis of a cognitive or memory-related disorder. In the case of COMPASS-31, the total score for participants with the diagnosis is 8.06 (SD = 1.93), and the total score for participants without the diagnosis is slightly higher, at 8.27 (SD = 1.78). Nevertheless, the outcome of the independent t-test (t = -0.963, p = 0.336) does not indicate a significant difference between the two groups. The effect size indicated by the Cohen's D value of -0.11 is very small, implying little practical importance. For CFQ specifically, the mean of the diagnosed is 80.6 (SD = 6.26), and the mean of the undiagnosed is 79.4 (SD = 6.89). The outcome of the t-test (t = 1.621, p = 0.106) shows that the difference is small, although not significant. The effect size, as indicated by the Cohen's d value of 0.19, is small, suggesting that there was no significant difference between the groups. Generally, these findings indicate that there are no significant differences in measures of OI or cognitive failure scores based on the formal diagnosis of a cognitive or memory-related disorder.

**Table 4:** Comparison among variables (formal diagnosis of cognitive- or memory-related condition) Independent t-test; p < 0.05 was considered statistically significant COMPASS-31: Composite Autonomic Symptom Score, CFQ: Cognitive Failure Questionnaire, SD: standard deviation, LL: lower limit, UL: upper limit, Cl: confidence interval

Variable	Yes (N = 282); mean ± SD	No (N = 103); mean ± SD	t	p	Cl 95% LL	UL	Cohen’s D
COMPASS-31	8.06 ± 1.93	8.27 ± 1.78	-0.963	0.336	-0.638	0.218	-0.11
CFQ	80.6 ± 6.26	79.4 ± 6.89	1.621	0.106	-0.256	2.656	0.19

Table 5 shows the comparison of COMPASS-31 and CFQ scores of the participants who were under medication (N = 245) and those who were not under medication (N = 140). In both measures, the two groups did not show a significant difference. The scores on COMPASS-31 were also very close (8.07 ± 1.90 on medication, 8.20 ± 1.87 without medication); the t-test yielded t = -0.649 (p = 0.517), and the effect size (Cohen's d) was -0.07, which is negligible. Likewise, CFQ scores were nearly the same (80.2 ± 6.62 with medication, 80.3 ± 6.17 without), with a t-test outcome of t = -0.146 (p = 0.884) and a Cohen's D of -0.02, a still smaller effect size. These results suggest that medications do not have a significant impact on OI and cognitive failure scores in this population.

**Table 5:** Comparison among variables (currently taking medication) Independent t-test; p < 0.05 was considered statistically significant COMPASS-31: Composite Autonomic Symptom Score, CFQ: Cognitive Failure Questionnaire, SD: standard deviation, LL: Lower limit, UL: Upper limit, Cl: confidence interval

Variable	Yes (N = 245); mean ± SD	No (N = 140); mean ± SD	t	p	Cl 95% LL	UL	Cohen’s D
COMPASS-31	8.07 ± 1.90	8.20 ± 1.87	-0.649	0.517	-0.524	0.264	-0.07
CFQ	80.2 ± 6.62	80.3 ± 6.17	-0.146	0.884	-1.445	1.245	-0.02

Table 6 shows the comparison of COMPASS-31 and CFQ scores of the patients who experienced the symptoms of OI (yes, N = 249), those who did not (no, N = 112), and those who were not certain (not sure, N = 24). In the case of COMPASS-31, mean scores were almost identical in all groups (8.14 ± 1.93 in the yes group, 8.11 ± 1.85 in the no group, and 7.92 ± 1.69 in the not sure group). A one-way ANOVA revealed a significant effect (p = 0.008), although the effect size (η² = 0.0008) was very small, which corresponds to a negligible practical effect. In the case of CFQ, the mean scores also did not differ markedly between the groups (80.3 ± 6.5 in the yes group, 80.0 ± 6.3 in the no group, and 80.3 ± 6.4 in the not sure group), and the difference was significant (p = 0.001), but the effect size was again small (η² = 0.004). These findings allow us to consider that, although the differences in both measures are statistically significant, the practical significance of these differences is negligible.

**Table 6:** Comparison of variables (symptoms of OI) One-way analysis of variance (ANOVA); p < 0.05 was considered statistically significant COMPASS-31: Composite Autonomic Symptom Score, CFQ: Cognitive Failure Questionnaire, SD: standard deviation, F: F-ratio, η2: effect size, OI: orthostatic intolerance

Variable	Yes (N = 249); mean ± SD	No (N = 112); mean ± SD	Not sure (N = 24); mean ± SD	p	F (2,382)	η2
COMPASS-31	8.14 ± 1.93	8.11 ± 1.85	7.92 ± 1.69	0.008	0.155	0.0008
CFQ	80.3 ± 6.5	80.0 ± 6.3	80.3 ± 6.4	0.001	0.077	0.004

**Figure 1:**
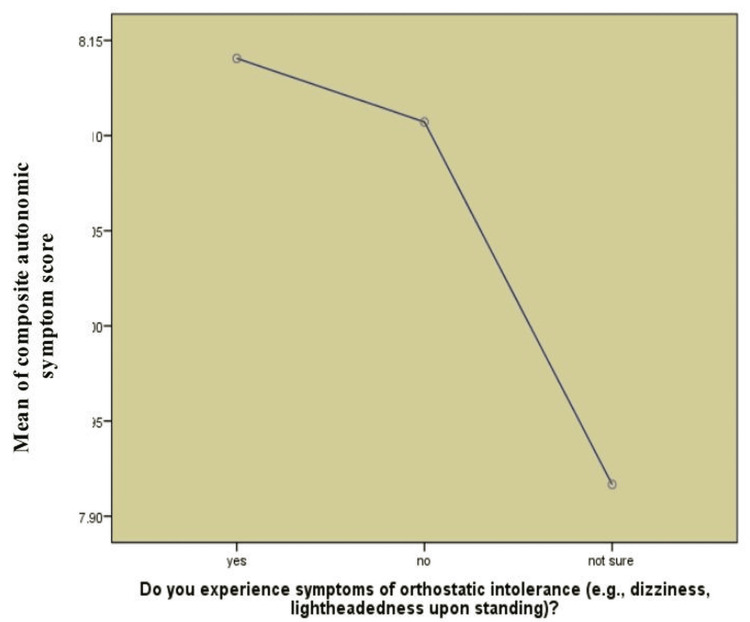
Association between OI symptoms and autonomic symptom severity OI: orthostatic intolerance

Figure 1 displays how average COMPASS-31 varied among people who reported dizziness or lightheadedness upon standing, those who did not, and those who were not sure (previously did or did not but are now unsure). Those with OI showed greater levels of autonomic symptoms on average (a mean composite score of 8.1 or higher) compared to the group who did not report the condition. A lower mean score in those who answered "no" suggests they experienced fewer autonomic symptoms. The fact that "not sure" responders had the lowest score on the mean indicates that they experienced either less awareness or a less severe problem with their autonomic nervous system. They suggest that experiencing orthostatic symptoms may be related to having severe autonomic dysfunction.

**Figure 2:**
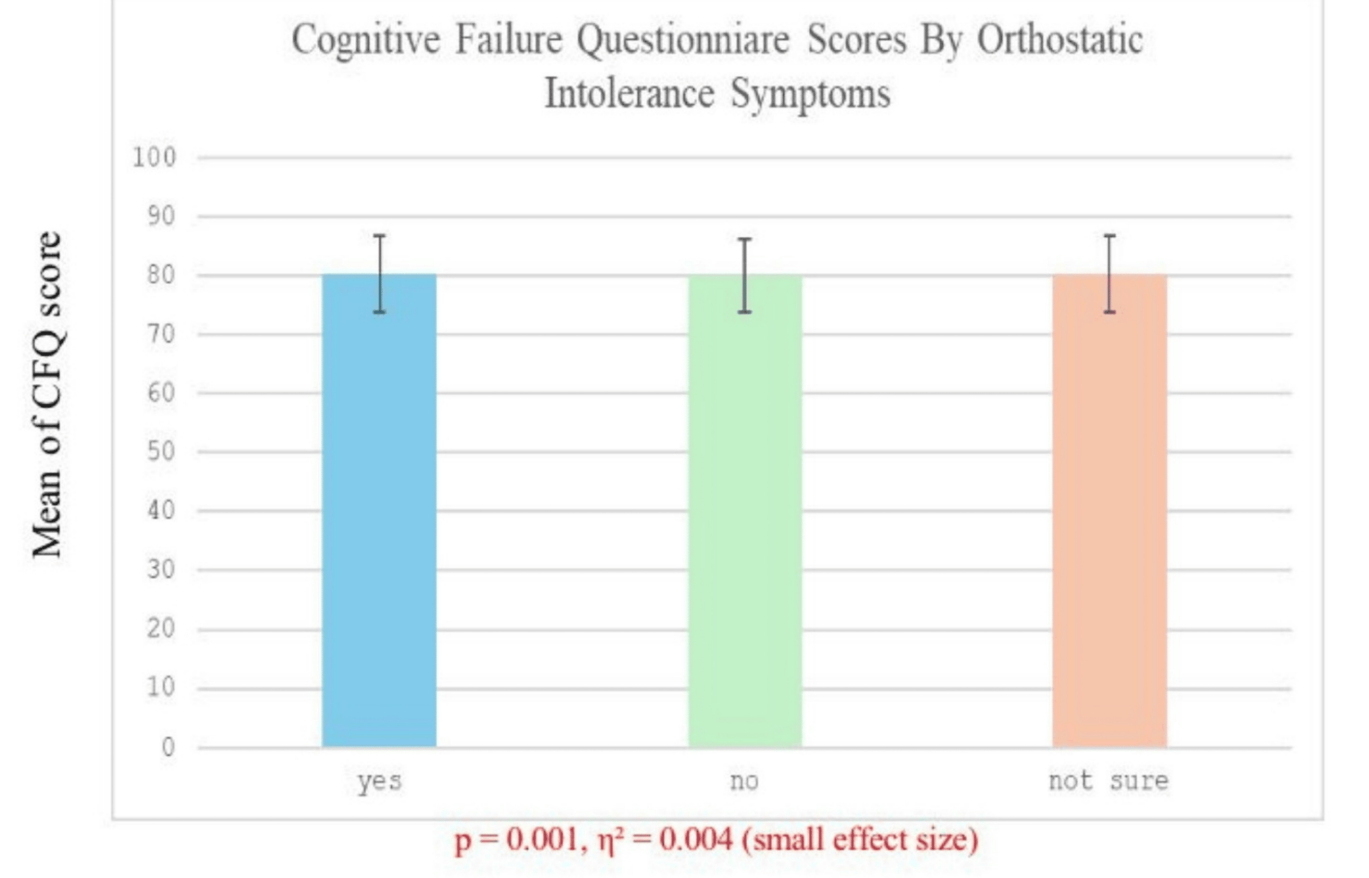
Relationship between OI and cognitive failures p < 0.05 was considered statistically significant, η2: effect size OI: orthostatic intolerance, CFQ: Cognitive Failure Questionnaire

Figure 2 demonstrates the connection between cognitive failures and OI. It compares CFQ scores among three categories: yes, no, and not sure. The yes group presents the highest CFQ scores, which refers to more cognitive failures. The statistics are significant (p = 0.001), with an insignificant effect size (η² = 0.004).

Table 7 illustrates the frequency with which individuals report experiencing brain fog in conjunction with other symptoms. Results from a one-way ANOVA indicated that the scores on COMPASS-31 were different in groups classified by brain fog frequency (p = 0.002, F(4, 380) = 5.742). Reporting frequent brain fog was linked to the lowest score (M = 7.54), while reporting brain fog either occasionally (M = 8.49) or rarely (M = 8.52) was associated with nearly the same score. Groups that stated "never" or "not sure" got results close to the average. The study found that a higher number of brain fog episodes is associated with lower scores of autonomic symptoms.

**Table 7:** Comparison of variables (symptoms of brain fog) One-way analysis of variance (ANOVA); p<0.05 was considered statistically significant COMPASS-31: Composite Autonomic Symptom Score, CFQ: Cognitive Failure Questionnaire, SD: standard deviation, F: F-ratio, η2: effect size

Variable	Yes, frequently (N = 74); mean ± SD	Yes, occasionally (N = 140); mean ± SD	Rarely (N = 129); mean ± SD	Never (N = 43); mean ± SD	Not sure (N = 43); mean ± SD	p	F (4,380)	η2
COMPASS-31	7.54 ± 1.77	8.48 ± 1.77	8.49 ± 1.99	8.38 ± 2.07	7.50 ± 1.92	0.002	5.742	-
CFQ	80.5 ± 7.3	80.2 ± 5.6	80.1 ± 6.4	79.7 ± 5.8	79.5 ± 8.1	<0.001	0.135	0.014

Even though the mean scores for CFQ were similar for each group (ranging from 79.5 to 80.5), the difference was examined as significant (p < 0.001) and small in effect (η² = 0.014). It suggests that brain fog symptoms influence how often individuals mention cognitive failures. Table 6 shows that there is a strong connection between experiencing brain fog frequently and having severe autonomic symptoms and cognitive concerns, but the links are moderate.

**Figure 3:**
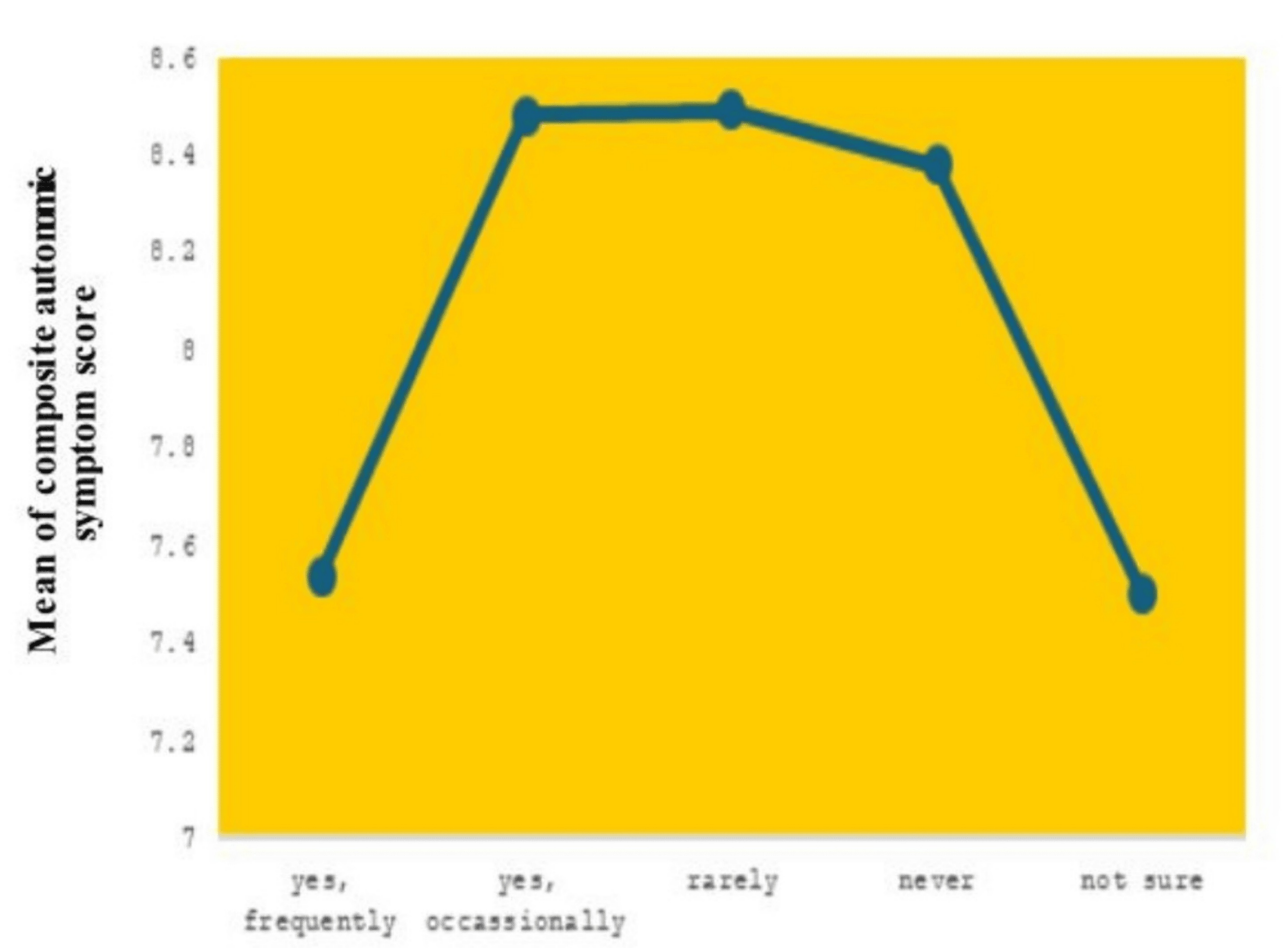
Autonomic symptom severity across frequencies of brain fog experience

Figure 3 displays the correlation between the severity of autonomic symptoms and brain fog. The numbers indicate that those with frequent or occasional brain fog experience a greater average COMPASS-31. This score is significantly lower among respondents who describe rarely or never experiencing the described phenomenon, with the lowest scores reported among those who are uncertain about their experiences with brain fog. The plot shows a definite positive trend of increased prevalence of brain fog, followed by a steep negative slope as the prevalence reduces.

**Figure 4:**
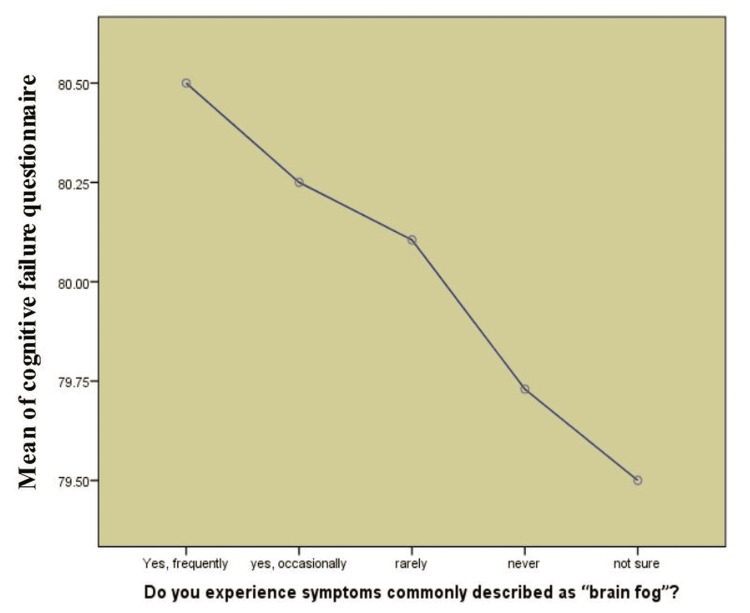
Cognitive failures and brain fog frequency

Figure 4 demonstrates that there is a clear link between having brain fog and scoring high on CFQ. Experiencing brain fog was strongly related to having more noticeable cognitive troubles, reflected by the highest CFQ scores (over 80.5). As people experienced brain fog no longer occurring, their mean CFQ scores fell, and the scores were lowest among those who said they did not experience brain fog at all. This implies that experiencing brain fog often can lead to people perceiving their mental abilities to be worse.

Table 8 demonstrates the findings of a linear regression model in which the CFQ score was modeled based on COMPASS-31. The constant (intercept) value was 81.498, and the 95 percent confidence interval (CI) of this value was 78.647-84.349. The p-value was <0.001, which shows that the constant value is significantly different from zero. COMPASS-31 presented a coefficient of 0.153 (95% CI: 0.495 to 0.189), a standard error of 0.174, and a standardized coefficient (B) of 0.449. The p-value of this predictor was 0.03, which implies that the OI subscale is a significant predictor of the CFQ score, but the effect is small. These findings suggest that OI, as evaluated using COMPASS-31, has a moderate impact on cognitive failure, as measured by CFQ.

**Table 8:** Linear regression analysis predicting CFQ score using COMPASS score ** p < 0.01 considered significant COMPASS-31: Composite Autonomic Symptom Score, CFQ: Cognitive Failure Questionnaire, B: coefficient, SE: standard error, β: standardized coefficient, LL: lower limit, UL: upper limit, Cl: confidence interval

Variable	B	95% Cl		SE	β	P
		LL	UL			
Constant	81.498	78.647	84.349	1.450	-	<0.001
COMPASS-31	0.153	0.495	0.189	0.174	0.449	0.03

**Figure 5:**
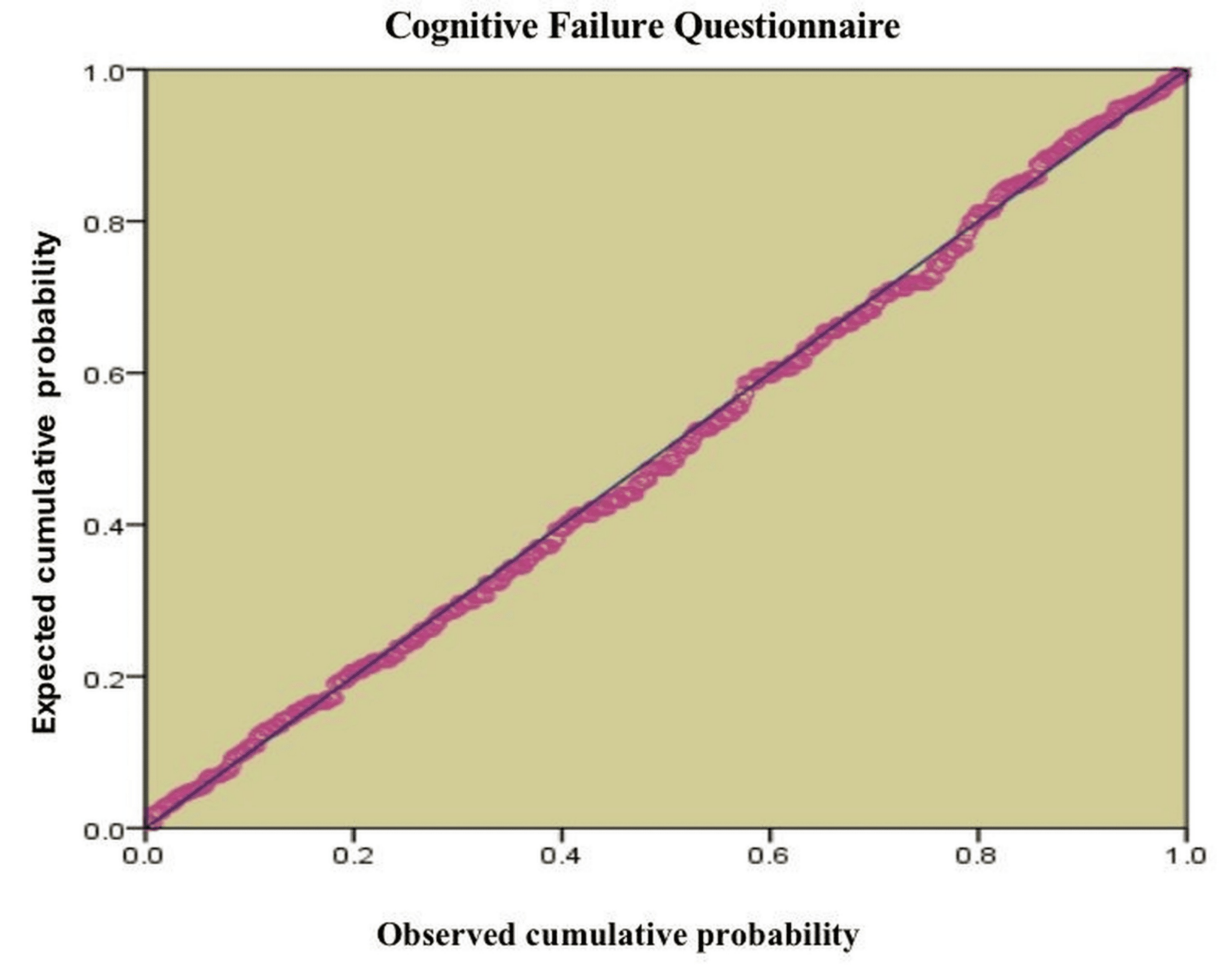
Normal P-P plot of standardized residuals for cognitive failures questionnaire scores

Figure 5 shows a normal P-P plot of the standardized residuals from the regression model for CFQ scores. The data points are closely in line with the diagonal reference line, which means that the residuals are nearly normally distributed. This pattern confirms the assumption of normality, which is one of the primary requirements for conducting valid parametric regression analysis. The diagonal alignment indicates that the model's errors are usually randomly distributed, thus increasing confidence in the accuracy and reliability of the regression findings. This assures that the regression model satisfies the condition of normally distributed residuals, making the statistical inferences obtained from the analysis valid.

Table 9 presents the breakdown of participants by age group, clinical diagnosis, and self-reporting of brain fog symptoms. Most participants were in the 18-24 age group (N = 344), followed by fewer in the under 18 (N = 14) and 25-34 (N = 27) age groups. Clinical diagnoses differed by age, with JHSD and hEDS being the most prevalent. A chi-square test revealed a statistically significant correlation between age category and clinical diagnosis (p = 0.04, χ² = 7.86), indicating that age affects the prevalence of diagnoses in this sample.

**Table 9:** Descriptive statistics of demographic variables (age, clinical diagnosis, and symptoms of brain fog) p-values calculated using the chi-square test; the significance level is set at p < 0.05 f: frequency, %: percentage, hEDS: hypermobile Ehlers-Danlos syndrome, JHSD: joint hypermobility spectrum disorder, POTS: postural orthostatic tachycardia syndrome, p: level of significance

Variables	f	hEDS	JHSD	Clinical diagnosis POTS	Another form of EDS	Not formally diagnosed	p	x^2^	Yes, frequently	Yes, occasionally	Symptoms of brain rarely	Never	Not sure	p	x^2^
Age	-	-	-	-	-	-	0.04	7.86	-	-	-	-	-	0.05	14.07
Under 18 years	14	6	3	4	0	1	-	-	2	6	3	2	1	-	-
18-24 years	344	111	151	57	15	10	-	-	131	115	63	32	3	-	-
25-34 years	27	7	16	3	1	0	-	-	7	7	10	3	0	-	-

Regarding brain fog symptoms, respondents reported varying frequencies of occurrence, with the highest numbers observed across all symptom categories in the 18-24 age group. The association between symptoms of brain fog and age was found to be significant as well (p = 0.05, χ² = 14.07), meaning that cognitive symptoms vary by age group.

Table 10 compares the occurrence of brain fog symptoms and diagnosis between people who have and those who do not have self-reported OI. Of the 249 people who mentioned OI, JHSD was reported in 107 cases, hEDS in 88, and POTS in 12. There were fewer cases of other EDS types and unclassified conditions. Yet, results from the chi-square test indicated that there was no meaningful link between clinical diagnosis and OI symptoms (p = 0.66, χ² = 5.849).

**Table 10:** Descriptive statistics of demographic variables (symptoms of OI, clinical diagnosis, and symptoms of brain fog) p-values calculated using the chi-square test; the significance level is set at p < 0.05 f = frequency, % = percentage, hEDS: hypermobile Ehlers-Danlos syndrome, JHSD: joint hypermobility spectrum Disorder, POTS: postural orthostatic tachycardia syndrome, p: level of significance, OI: orthostatic intolerance

Variables	f	hEDS	JHSD	Clinical diagnosis POTS	Another form of EDS	Not formally diagnosed	p	x^2^	Yes, frequently	Yes, occasionally	Symptoms of brain fog rarely	Never	Not sure	p	x^2^
Symptoms of OI	-	-	-	-	-	-	0.66	5.849	-	-	-	-	-	0.005	6.567
Yes	249	88	107	40	8	6	-	-	83	85	53	26	2	-	-
No	112	30	53	18	7	4	-	-	49	35	18	9	1	-	-
Not sure	24	6	10	6	1	1	-	-	8	8	5	2	1	-	-

There was also a statistically significant connection found between OI symptoms and the experience of brain fog (p = 0.005, χ² = 6.567). In people with OI, the number of complaints about brain fog (either frequent or occasional) was higher than in those without OI. This means that OI may not be linked to any specific diagnosis, but it is often associated with more complaints about the mind, such as brain fog.

## Discussion

This research investigated the connection between OI symptoms and cognitive symptoms in people diagnosed with hEDS and JHSD. Our findings indicate a correlation between OI symptoms and cognitive failures, aligning with research that has illustrated that patients who have autonomic failure have impaired executive function under orthostatic stress, believed to be the result of transient brain hypoperfusion. This confirms the association between autonomic dysfunction and cognitive impairment with orthostatic stress [21].

We found that the OI scores were not significantly different between those with and without a cognitive diagnosis, which is consistent with the literature, which mentions that OH is frequently under-recognized because it presents in a nonspecific or subclinical fashion. This suggests that the autonomic symptoms can exist with or without a formal diagnosis [22]. In our study, patients with clinically diagnosed cognitive failure had a slightly higher score, but the difference was not statistically significant. This aligns with earlier reports, which had highlighted the challenge of distinguishing between actual cognitive complaints and confounding psychiatric or neurological conditions [23].

We found that the advantage of medication use in reducing orthostatic symptoms is not significant, which is in line with other reviews demonstrating a low level of evidence supporting most of the recommended interventions, including salt-loading therapy. Further clinical testing is, however, necessary, as we did not conduct bedside orthostatic testing to ensure that all symptoms were directly attributable to OH or postural tachycardia. In combination with the above, these results underscore the need for more definitive clinical trials to evaluate the long-term efficacy of current OI treatment approaches [24]. We did not find any significant difference in the scores of cognitive failure between the medicated and unmedicated participants in our study, probably because we excluded the people with psychiatric or neurological disorders. Nevertheless, evidence-based practice among previous researchers indicated that psychotropic medication can negatively affect cognition, especially in people with a history of mental illness [25].

Our results reveal a statistically significant but weak correlation between subjective cognitive complaints and OI symptoms. This aligns with earlier population-based studies suggesting that although orthostatic symptoms could heighten the risk for mild cognitive impairment, the correlation is modest and moderated by factors like hypertension and age. Therefore, OI has a subtle effect on cognition rather than a strong or direct influence [26].

We observed that recurrent brain fog was associated with worse autonomic symptoms, but the relationship was not linear. Whereas the severity of autonomic symptoms increased with more frequent brain fog reports, the association was more complex and differed by brain fog frequency. This is consistent with results that indicate that the brain fog severity is likely to be associated with conditions such as neuropathic POTS. However, it is worth noting that the severity of symptoms does not necessarily correlate with objective autonomic dysfunction, as assessed by autonomic reflex tests [27]. Consistent with our results, post-COVID-19 research has also identified that individuals experiencing frequent brain fog mostly encounter difficulties related to attention and memory, which are significantly associated with subjective cognitive complaints as well as fatigue [28]. This is in support of the fact that brain fog is affected by both physiological (e.g., autonomic dysfunction) and psychological (e.g., depression, fatigue) factors [28].

Our results are consistent with previous research indicating that autonomic dysfunction is linked with cognitive impairment. For example, parasympathetic deficits have been reported in people with mild cognitive impairment and are related to impaired neuropsychological functioning. This agreement aligns with the notion that autonomic symptoms, such as OI, may serve as early indicators of cognitive vulnerability or decline [29].

Our research demonstrates that most participants with hEDS or JHSD diagnoses were young adults, predominantly within the 18- to 24-year bracket. Conversely, previous literature predominantly discusses symptom development and clinical issues in older adults with hEDS or JHSD [30]. This imbalance highlights a significant gap in understanding the entire clinical lifecycle of hEDS or JHSD throughout the lifespan. It emphasizes the necessity for additional research centered on aging in this population [30]. Our results indicated that the symptoms of brain fog differed remarkably by age, with the highest prevalence occurring in young individuals. This was consistent with previous work in 14- to 29-year-old POTS patients, in which brain fog was similarly prevalent and associated with cognitive impairment provoked by orthostatic stress and fatigue, emphasizing autonomic function as a key mechanism for cognitive symptoms [31].

Our results demonstrate a strong correlation between brain fog symptoms and OI symptoms, independent of individual clinical diagnosis. This confirms the emerging insight that autonomic dysfunction is commonly found in conjunction with cognitive dysfunction, likely mediated by multiple complex mechanisms, including immune and vascular dysregulation. These findings underscore the utility of assessing autonomic symptoms in the treatment of cognitive complaints in varied patient populations [32].

These findings have significant clinical implications. First, they emphasize the importance of thorough autonomic evaluation in patients with hypermobility disorders who report cognitive difficulties. Second, they suggest that treating autonomic symptoms can alleviate some of the cognitive impairments experienced by this group. Future studies should investigate whether interventions specifically targeting OI-volume expansion, pharmacotherapy, or physical countermeasures can lead to quantifiable improvements in cognitive ability.

Limitations

There are some limitations to be mentioned. The cross-sectional nature of the study precludes causal inference. However, an association between OI symptoms and complaints of cognitive dysfunction was found; we cannot determine the direction of this association. Moreover, the use of self-report questionnaires, though validated, is susceptible to recall bias or mood symptom-related influence. More objective determination of cognitive function and autonomic variables (e.g., tilt-table testing, neuropsychological testing) would enhance future research. Lastly, while the sample size was sufficient for the analyses performed, bigger multicenter trials will be necessary to extrapolate these findings to the broader hEDS or JHSD population.

Future directions

Future studies can extend the findings using longitudinal designs to establish the causal association between OI and cognitive complaints in patients with hEDS and JHSD. The incorporation of objective assessments, such as tilt-table testing, neuropsychological tests, and brain imaging modalities like functional MRI, may elucidate the physiological and neurological basis of cognitive dysfunction associated with OI. Furthermore, intervention studies are needed to determine whether therapies targeting autonomic symptoms improve cognitive outcomes. Future research should also examine the contribution of coexisting factors like fatigue, anxiety, depression, and sleep disturbances, which might underlie the perception of cognitive impairment. Enrolling larger and more representative samples would enable subgroup analysis and increase the generalizability of the findings. Investigating younger groups can also help identify early risk indicators and inform early intervention approaches.

## Conclusions

This research shows a significant association between symptoms of OI and cognitive symptoms, especially brain fog, in patients with hEDS and JHSD. These findings underscore the clinical significance of autonomic dysfunction in cognitive symptomatology, suggesting that the cognitive symptoms in this population may have a physiological basis through autonomic dysfunction rather than being purely psychological. Although the correlations are weak, they are consistent with the developing literature and document the complexity of symptom interplay in hEDS and JHSD. Clinicians treating cognitive complaints should also consider assessing and managing autonomic symptoms in patients with hypermobility-related disorders. Research studies that incorporate objective physiological and cognitive measurements to determine the effectiveness of targeted interventions are needed to further our understanding and treatment of this understudied patient group.
